# Skn-1a/Pou2f3 is required for the generation of Trpm5-expressing microvillous cells in the mouse main olfactory epithelium

**DOI:** 10.1186/1471-2202-15-13

**Published:** 2014-01-16

**Authors:** Tatsuya Yamaguchi, Junpei Yamashita, Makoto Ohmoto, Imad Aoudé, Tatsuya Ogura, Wangmei Luo, Alexander A Bachmanov, Weihong Lin, Ichiro Matsumoto, Junji Hirota

**Affiliations:** 1 Department of Bioengineering, Graduate School of Bioscience and Bioengineering, Tokyo Institute of Technology, Yokohama 226-8501, Japan; 2 Monell Chemical Senses Center, 3500 Market Street, Philadelphia, PA 19104, USA; 3 Department of Biological Sciences, University of Maryland, Baltimore County, Baltimore, MD 21250, USA; 4 Center for Biological Resources and Informatics, Tokyo Institute of Technology, 4259-B63 Nagatsuta-cho, Midori-ku, Yokohama 226-8501, Japan

## Abstract

**Background:**

The main olfactory epithelium (MOE) in mammals is a specialized organ to detect odorous molecules in the external environment. The MOE consists of four types of cells: olfactory sensory neurons, supporting cells, basal cells, and microvillous cells. Among these, development and function of microvillous cells remain largely unknown. Recent studies have shown that a population of microvillous cells expresses the monovalent cation channel Trpm5 (transient receptor potential channel M5). To examine functional differentiation of Trpm5*-*expressing microvillous cells in the MOE, we investigated the expression and function of Skn-1a, a POU (Pit-Oct-Unc) transcription factor required for functional differentiation of Trpm5*-*expressing sweet, umami, and bitter taste bud cells in oropharyngeal epithelium and solitary chemosensory cells in nasal respiratory epithelium.

**Results:**

*Skn-1a* is expressed in a subset of basal cells and apical non-neuronal cells in the MOE of embryonic and adult mice. Two-color *in situ* hybridization revealed that a small population of *Skn-1a*-expressing cells was co-labeled with *Mash1/Ascl1* and that most *Skn-1a-*expressing cells coexpress *Trpm5*. To investigate whether Skn-1a has an irreplaceable role in the MOE, we analyzed *Skn-1a-*deficient mice. In the absence of Skn-1a, olfactory sensory neurons differentiate normally except for a limited defect in terminal differentiation in ectoturbinate 2 of some of MOEs examined. In contrast, the impact of Skn-1a deficiency on Trpm5*-*expressing microvillous cells is much more striking: Trpm5, villin, and choline acetyltransferase, cell markers previously shown to identify Trpm5-expressing microvillous cells, were no longer detectable in *Skn-1a-*deficient mice. In addition, quantitative analysis demonstrated that the density of superficial microvillous cells was significantly decreased in *Skn-1a*-deficient mice.

**Conclusion:**

*Skn-1a* is expressed in a minority of *Mash1-*positive olfactory progenitor cells and a majority of Trpm5-expressing microvillous cells in the main olfactory epithelium. Loss-of-function mutation of *Skn-1a* resulted in complete loss of Trpm5-expressing microvillous cells, whereas most of olfactory sensory neurons differentiated normally. Thus, Skn-1a is a critical regulator for the generation of Trpm5-expressing microvillous cells in the main olfactory epithelium in mice.

## Background

A sense of smell is essential for the survival of both individuals and species. The main olfactory epithelium (MOE) is considered to be responsible for detecting a vast number of airborne odorous chemicals. The MOE consists of four major types of cells: olfactory sensory neurons (OSNs), supporting cells, basal cells, and microvillous cells [1]. The OSNs are ciliated bipolar neurons specialized in detecting odorants and send their information to the axonal target in the main olfactory bulb. The cell bodies of the terminally differentiated OSNs are located in the intermediate position of the MOE. The supporting cells, also called sustentacular cells, protect and support OSNs, much like glial cells in the central nervous system. The supporting cells span the entire basal to apical extent of the MOE, and their somata are located in the apical/superficial layer of the MOE. The basal cells, which are globose and horizontal cells, are considered to function as stem cells that give rise to OSNs and supporting cells.

Although the properties of OSNs, supporting cells, and basal cells have been well studied and characterized in terms of both development and function, those of the microvillous cells remain largely unknown in the MOE. Microvillous cells are less abundant than are OSNs and supporting cells and are scattered in the superficial layer of the MOE [2-5]. Morphologically, at least three different types of microvillous cells have been described [3]. Two of them express the monovalent cation channel transient receptor potential channel M5 (Trpm5). Because Trpm5 plays a critical role in chemical sensing in sweet, umami, and bitter taste cells (so-called type II taste cells) and in solitary chemosensory cells (SCCs) [6-10], and because the chemosensory activities of these taste cells are Trpm5-dependent and thermosensitive [11], Trpm5-expressing microvillous cells (Trpm5-microvillous cells) in the MOE are considered to be chemo- and/or thermosensitive. Indeed, Trpm5-microvillous cells were shown to express choline acetyltransferase (ChAT) and the vesicular acetylcholine transporter, to respond to chemical or thermal stimuli, and to release acetylcholine to modulate activities of neighboring supporting cells and OSNs [12]. However, molecular mechanisms underlying the generation and differentiation of these cells are not well understood.

Skn-1a (also known as Pou2f3), a POU (Pit-Oct-Unc) transcription factor, is expressed in *Trpm5-*expressing chemosensory cells: type II taste cells in taste buds of oropharyngeal epithelium and SCCs in nasal respiratory epithelium. Its loss-of-function mutation resulted in defective generation and/or functional differentiation of type II taste cells and SCCs [13,14]. Thus, Skn-1a functions as a determinant for the generation and functional differentiation of these cells. Here we show that *Skn-1a* is expressed in the MOE, where neither taste cells nor SCCs have been observed. We characterized *Skn-1a*-expressing cells and investigated the function of Skn-1a in the MOE using *Skn-1a*-deficient mice. We demonstrate that Skn-1a is necessary for the generation of Trpm5-microvillous cells.

## Results

### The expression of *Skn-1a* in the main olfactory epithelium

**Figure 1:**
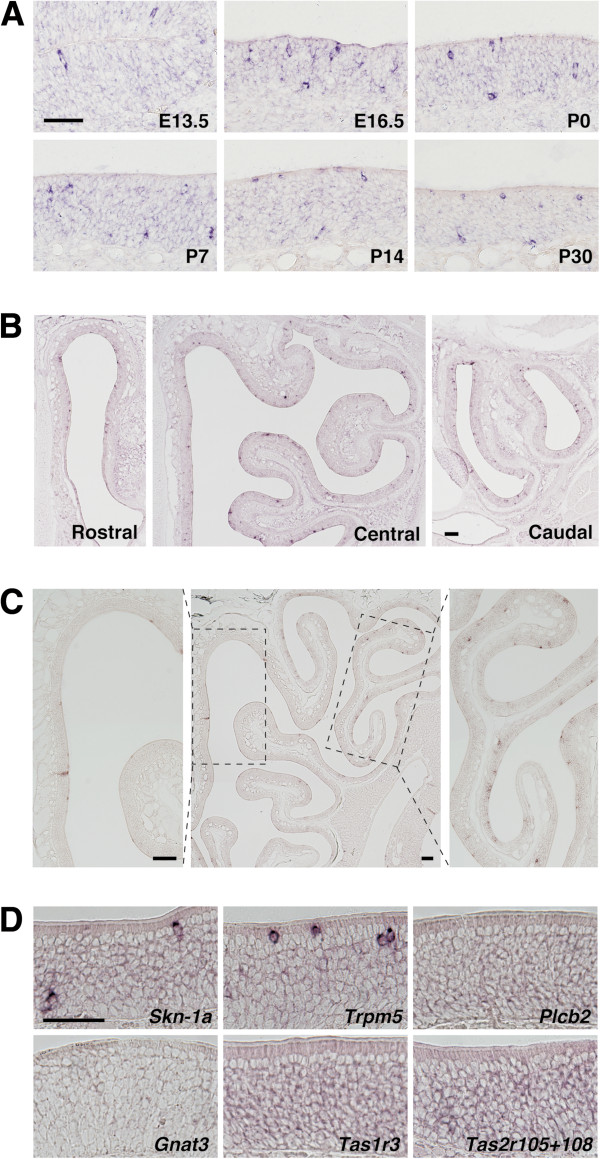
**Expression of *Skn-1a *in the developing main olfactory epithelia. (A)***In situ* hybridization with RNA probes for *Skn-1a* in coronal sections of mouse MOE at embryonic days 13.5 and 16.5 and postnatal days 0, 7, 14, and 30. The expression of *Skn-1a* was first detected at embryonic day 13.5 and was observed during subsequent development. The *Skn-1a*-expressing cells were located in apical, intermediate, and basal positions in the MOE during embryonic stages and were gradually restricted to apical and basal positions in postnatal development. **(B)** The expression of *Skn-1a* in the rostral-caudal axis of the MOE at postnatal day 7. *Skn-1a* expression was observed throughout the MOE, in terms of the rostral-caudal and the dorsal-ventral axis. **(C)** In the adult MOE, *Skn-1a*-expressing cells were distributed in graded fashion: low density in the dorsomedial region to high density in the lateral region. Left and right images are higher-magnification images of the dorsomedial and lateral regions (the areas enclosed by the dashed boxes in the center image), respectively. **(D)***In situ* hybridization of signaling molecules in SCCs on coronal sections of adult MOE. Expression of *Tas1r3*, *Tas2r105*, *Tas2r108*, *Gnat3*, and *Plcb2* was not observed. Only the signal of *Trpm5* mRNA was detected in the superficial layer of the MOE. Scale bars: 50 μm in A and D, 500 μm in B and C.

We previously demonstrated that *Skn-1a* is expressed in SCCs in nasal respiratory epithelium [14]. During expression analyses of *Skn-1a* in the nasal cavity, we noticed that *Skn-1a* mRNA signals were also observed in the MOE. Because Skn-1a is a crucial factor for the generation and/or functional differentiation of chemosensory cells such as sweet, umami, and bitter taste cells and SCCs, we hypothesized that Skn-1a could be involved in the generation of a certain cell type comprised in the MOE. First, we characterized *Skn-1a*-expressing cells in the MOE. *In situ* hybridization analyses revealed that the scattered signals of *Skn-1a* mRNA were first detectable at embryonic day 13.5 (Figure 1A). *Skn-1a-*expressing cells were located in apical, intermediate, and basal positions of the MOE during embryonic stages and were gradually restricted to apical and basal positions during postnatal development. We also analyzed the distribution of *Skn-1a*-expressing cells along the rostral-caudal axis and found scattered *Skn-1a* expression throughout the MOE at postnatal day 7 (Figure 1B). The distribution of *Skn-1a*-expressing cells in the dorso-ventral and the medial-lateral axis were uniform during embryonic and early postnatal stages, and shifted to a graded pattern in the adult MOE: smaller number of *Skn-1a*-expressing cells in the dorsomedial region and larger number in the ventrolateral region (Figure 1C).

To our knowledge, neither SCCs nor taste cells have been found in the MOE. Both cell types share expression of *Tas1r3*, Tas2r family genes, *Gnat3* (gustducin), *Plcb2*, and *Trpm5*. We examined the mRNA expression of these genes in the MOE and detected only *Trpm5* (Figure 1D). The *Trpm5* mRNA signals were observed in the superficial layer, where *Skn-1a* mRNA expression was also observed, indicating a possible role of Skn-1a in generation of cells of the MOE.

**Figure 2:**
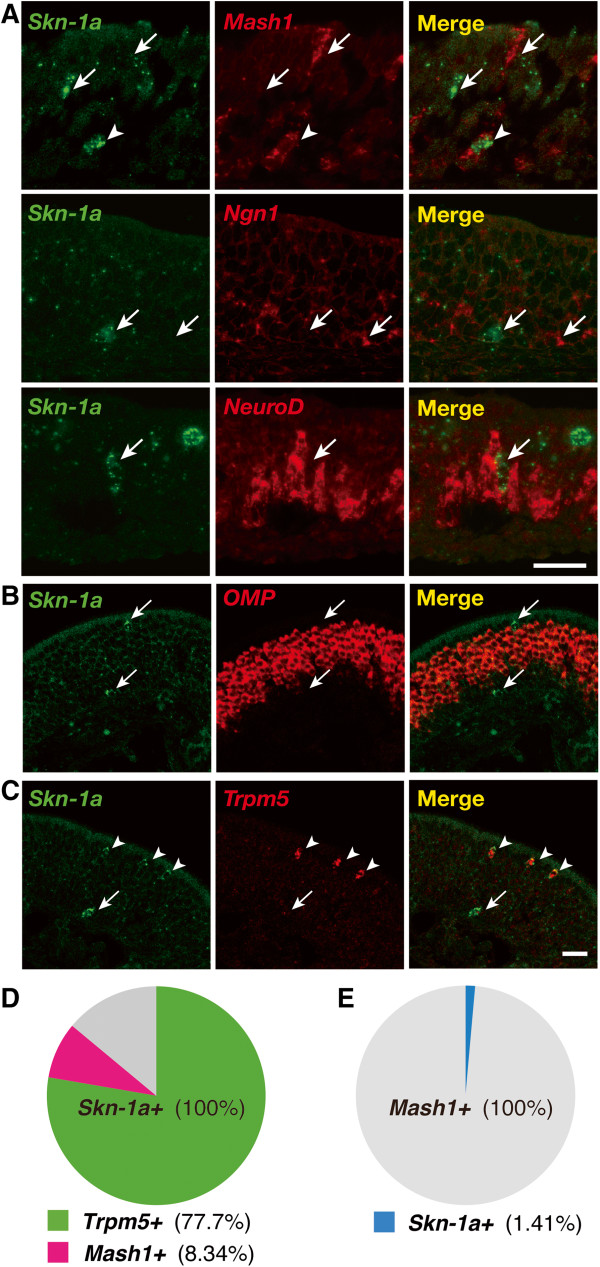
**Characterization of *Skn-1a*-expressing cells in the main olfactory epithelium. (A)***Skn-1a*-expressing cells were characterized using two-color *in situ* hybridization in coronal sections of the MOE at postnatal day 0 with RNA probes for *Skn-1a* (green) and OSN progenitor/precursor genes *Mash1* (neuronal progenitors), *Ngn1* (neuronal precursors), and *NeuroD* (differentiating/postmitotic neurons). Small populations of *Skn-1a*-potitive cells and *Mash1*-positive cells overlapped. The arrowhead indicates a co-labeled cell, and arrows indicate either *Skn-1a* or *Mash1* single-labeled cells*.* None of *Skn-1a*-positive cells were co-labeled with *Ngn1* and *NeuroD* (arrows). **(B and C)***In situ* hybridization of *Skn-1a* (green) with *OMP* (mature OSNs; B, red) and *Trpm5* (*Trpm5*-positive microvillous cells; C, red) in coronal sections of the adult MOE. Neither apical nor basal *Skn-1a*-expressing cells (arrows) were co-labeled with *OMP* signals. *Trpm5* signals were co-labeled with apical *Skn-1a* signals (arrowheads) but not with basal *Skn-1a* signals (arrow). Scale bars, 25 μm. **(D and E)** Populations of *Skn-1a-*expressing cells **(D)** and *Mash1-*expressing cells **(E)** were analyzed by two-color *in situ* hybridization at postnatal day 30. Quantitative analyses revealed that 8.34 ± 2.82% (mean ± SD) of the *Skn-1a-*expressing cells coexpressed *Mash1* (n = 3)*,* and 77.7 ± 5.95% coexpressed *Trpm5* (n = 3). In the OSN-lineage, *Mash1-*positive olfactory progenitors rarely expressed *Skn-1a* (1.41 ± 0.564%, n = 3).

To characterize *Skn-1a*-expressing cells in the MOE, we performed two-color *in situ* hybridization using riboprobes for specific molecular markers. Because of the scattered expression of *Skn-1a* in basal and apical positions of the adult MOE, *Skn-1a-*positive cells were considered to be neither terminal differentiated OSNs nor supporting cells, which occupy the intermediate and apical regions of MOE, respectively. But they could be basal OSN progenitor/precursor cells and/or apical microvillous cells. We first tested coexpression with basally located olfactory neuronal markers: *Mash1/Ascl1* (neuronal progenitors), *Ngn1/Neurog1* (neuronal precursors), and *NeuroD* (differentiating/postmitotic neurons). A small population of *Skn-1a*-potitive cells was co-labeled with *Mash1*, but no *Skn-1a*-positive cells were co-labeled with *Ngn1* or *NeuroD* (Figure 2A), indicating that *Skn-1a* is transiently expressed in some OSN progenitors, turning off prior to the subsequent precursor stage. As expected, *Skn-1a*-expressing cells were not co-labeled with *OMP*, a marker for mature OSNs (Figure 2B). We then examined the relationship of the expression of *Skn-1a* with *Trpm5* and found that *Skn-1a*-expressing cells in the apical position were co-labeled with *Trpm5* (Figure 2C).

To analyze the population of *Skn-1a-*expressing cells, we performed two-color *in situ* hybridization for *Skn-1a* in combination with either *Mash1* or *Trpm5* and counted the number of single- and double-positive cells at postnatal day 30 (Figure 2D). Because of total number of cells counted differed between sections due to the scattered expression of *Skn-1a* and *Trpm5*, we represent the population of *Skn-1a-*expressing cells in percentage. Quantitative analyses revealed that 8.34 ± 2.82% (mean ± SD) of the *Skn-1a-*expressing cells were *Mash1* positive (n = 3, see Additional file 1: Table S1) and 77.7 ± 5.95% were *Trpm5* positive (n = 3, see Additional file 1: Table S1). Thus, a large population of *Skn-1a*-expressing cells is *Trpm5* positive, and *Mash1-*positive cells are a minor population. In the OSN lineage, *Mash1-*positive olfactory progenitors rarely expressed *Skn-1a* (1.41 ± 0.564%, n = 3, see Additional file 1: Table S1; Figure 2E), whereas 36.9 ± 15.0% of *Trpm5-*expressing cells coexpress *Skn-1a* (n = 3, see Additional file 1: Table S1)*.* These results suggest involvement of Skn-1a in the Trpm5*-*microvillous cell lineage rather than in the OSN lineage.

### Impact of *Skn-1a* deficiency on olfactory sensory neuronal lineage

**Figure 3:**
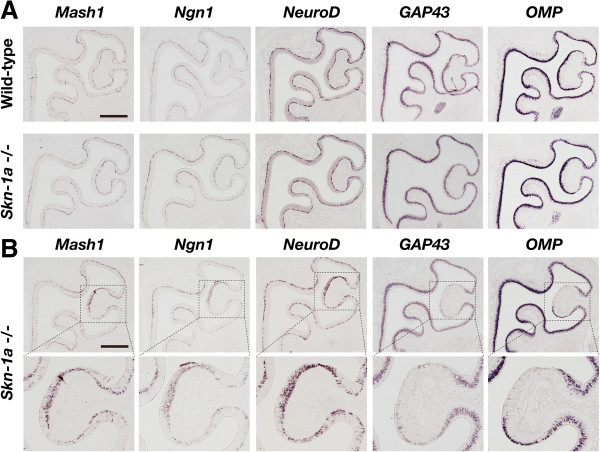
**Effect of *Skn-1a *deficiency on the differentiation of olfactory sensory neurons.** The impact of Skn-1a deficiency on the OSN differentiation was examined by *in situ* hybridization using OSN neuronal marker genes *Mash1* (neuronal progenitors), *Ngn1* (neuronal precursors), *NeuroD* (differentiating/postmitotic neurons), *GAP43* (immature neurons), and *OMP* (mature neurons) in coronal sections of wild-type and *Skn-1a*^-/-^ mice at postnatal day 7. **(A)** No obvious differences in the expression of marker genes were observed between *Skn-1a*^-/-^ and wild-type mice in most cases. **(B)** Examples of the *Skn-1a*^-/-^ mice showing a partial but obvious phenotype of a defective differentiation of OSNs only in the specific region of ectoturbinate 2 at postnatal day 7 (upper panels). Expression of *GAP43* and *OMP* was greatly suppressed, whereas expression of *Mash1, Ngn1,* and *NeuroD* was upregulated (lower panels: high magnification images of the dotted boxes). Scale bars, 500 μm.

Coexpression analyses revealed that *Skn-1a* is expressed in both OSN and Trpm5*-*microvillous cell lineages. To investigate the function of Skn-1a in these cell lineages, we analyzed *Skn-1a*-deficient mice. We first examined the impact of loss of Skn-1a function on differentiation of OSNs by *in situ* hybridization using RNA probes for neuronal marker genes *Mash1*, *Ngn1*, *NeuroD*, *GAP43* (immature OSNs), and *OMP*. The expression of all marker genes in *Skn-1a*^-/-^ mice was basically the same as in wild-type mice (Figure 3A). However, some *Skn-1a*^-/-^ mice showed a partial and limited phenotype of defective differentiation of OSNs, in which the expression of *GAP43* and *OMP* was greatly downregulated but expression of *Mash1*, *Ngn1*, and *NeuroD* was upregulated. This impaired terminal differentiation into OSNs was restricted in the area of ectoturbinate 2. Among five mice analyzed, we found this region-specific phenotype bilaterally (right and left MOE) in one mouse, unilaterally in two, and not at all in two. When we counted the right and left MOE separately (i.e., two MOE per mouse), the penetrance of this phenotype could be calculated as 40% (4 of 10 half MOEs). Because most of the MOE developed grossly normal and incomplete development of OSNs was observed partially in terms of both number and area of the *Skn-1a*^-/-^ MOE, we conclude that Skn-1a is not critical for the generation of OSNs in most MOE regions.

**Figure 4:**
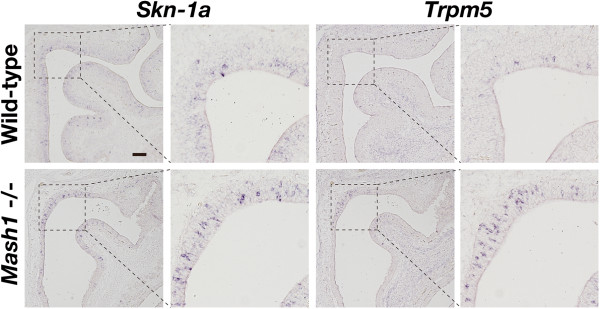
**Expression of *Skn-1a *and *Trpm5 *in the MOE of *Mash1***^**-/- **^**embryos.** Expression of *Skn-1a* and *Trpm5* in the *Mash1*^-/-^ MOE was examined by *in situ* hybridization at embryonic day 18.5. The MOE of *Mash1*^-/-^ embryos appeared smaller and thinner than that of wild-type littermates, as observed previously. Expression of either *Skn-1a* or *Trpm5* was observed in both the wild-type and *Mash1*^-/-^ MOE. Higher-magnification images of the dotted boxes are presented to the right of each image. Scale bars, 100 μm.

In the MOE, Mash1 functions as a determinant factor to generate OSNs. Because a small population of *Skn-1a*-expressing cells coexpress *Mash1,* it is conceivable that Mash1 affects Skn-1a function and/or Trpm5-microvillous cell lineage. To test this possibility, we also investigated the impact of Mash1 deficiency on *Skn-1a*-expressing cells by analyzing *Mash1*^-/-^ embryos at embryonic day 18.5. *In situ* hybridization showed expression of both *Skn-1a* and *Trpm5* in both wild-type and *Mash1*^-/-^ mice MOE (Figure 4), indicating that *Mash1* is not a determinant gene for Trpm5-microvillous cell lineage. Although the number of *Skn-1a/Trpm5*-coexpressing cells per unit area tended to increase in *Mash1*^-/-^ MOE, it is not clear if this was caused by a direct effect of the loss of function of Mash1. Because loss of Mash1 function causes severe malformation of the MOE and massive loss of OSNs, it is possible that the apparent increased number of *Skn-1a*/*Trpm5*-coexpressing cells is due to relative effects arising from a small MOE in the mutant.

### Impact of *Skn-1a* deficiency on Trpm5-positive microvillous cells

**Figure 5:**
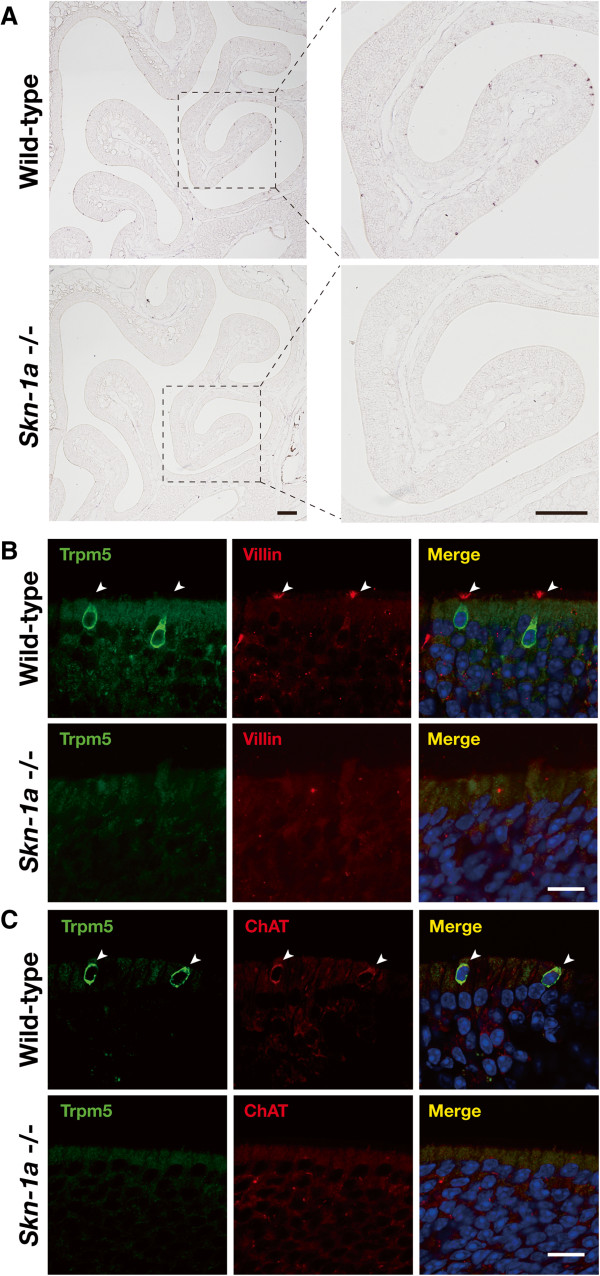
**Effect of *Skn-1a *deficiency on the functional differentiation of Trpm5-positive microvillous cell. (A)***In situ* hybridization of *Trpm5* on coronal sections of the MOE of wild-type and *Skn-1a*^-/-^ mice. The mRNA signal of *Trpm5* was absent in *Skn-1a*^-/-^ mice. (B and C) Coronal sections of wild-type and *Skn-1a*^-/-^ MOE of adult mice were immunostained with an anti-Trpm5 antibody (green) and an anti-villin **(B)** or anti-ChAT **(C)** antibody (red). Trpm5-positive cells were villin positive in the microvilli in the wild-type MOE (arrowheads), whereas no immunoreactive signal for Trpm5 or villin was observed in the *Skn-1a*^-/-^ MOE. Trpm5-positive cells were co-immunostained with anti-ChAT antibody in wild-type (arrowheads) but not in *Skn-1a*^-/-^ mice. Scale bars: 100 μm in A, 10 μm in B and C.

Because *Skn-1a* is expressed in Trpm5-microvillous cells, we next examined the impact of *Skn-1a* deficiency on Trpm5-microvillous cells. *In situ* hybridization revealed that the expression of *Trpm5* was completely absent in *Skn-1a*^-/-^ MOE (Figure 5A). We then analyzed the expression of markers for Trpm5-microvillous cells in *Skn-1a*^-/-^ mice by double-label immunostaining against Trpm5 combined with villin, a marker for microvilli, and ChAT. No immunoreactivity to villin or ChAT was detected in *Skn-1a*^-/-^ mice, but immunoreactivity was observed in Trpm5-positive cells in the MOE of wild-type mice (Figure 5B, C). These results suggest that Skn-1a is required for functional differentiation of Trpm5-microvillous cells, including the expression of Trpm5, villin, and ChAT, or the generation of Trpm5-microvillous cells.

### Quantitative comparison of *ChAT*/*Trpm5*-expressing microvillous cell density in *Skn-1a*^-/-^ and *ChAT-eGFP* mice

**Figure 6:**
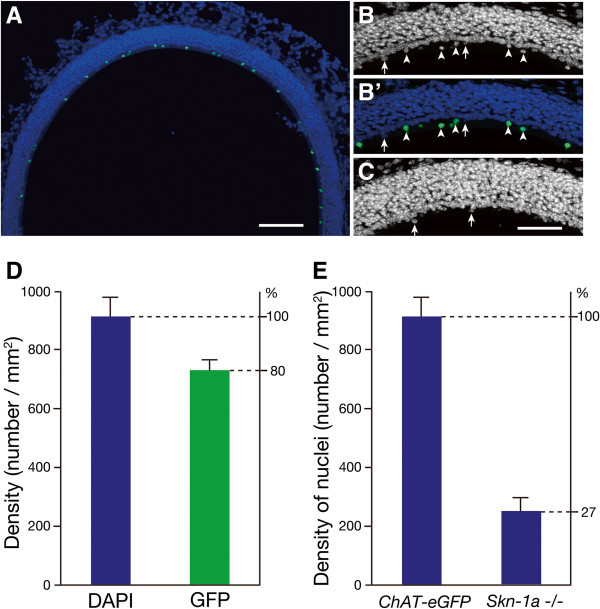
**Quantification of microvillous cell density in the most superficial layer of the MOE. (A)** Image of an MOE dorsal recess from a *ChAT-eGFP* mouse, showing *ChAT*/*Trpm5*-expressing microvillous cells (GFP^+^) in the most superficial layer, a region above the supporting cell nuclei. **(B)** A higher-magnification view of the DAPI-stained nuclei in the dorsal MOE. Arrowheads point to nuclei of GFP^+^ microvillous cells. (B’) Overlay of GFP signal onto B. **(C)** Image of an MOE dorsal recess from an *Skn-1a*^-/-^ mouse. Arrows in B and C point to nuclei that do not belong to GFP^+^ microvillous cells. **(D)** Plot of the averaged density per surface area of DAPI-stained nuclei and GFP^+^ cells in the most superficial layer of the MOE from *ChAT-eGFP* mice. Counting was conducted from the dorsal recess and septum of the MOE. Approximately 80% of the cells in the area are GFP^+^ microvillous cells. **(E)** Comparison of averaged nucleus density, showing approximately 73% reduction in the nucleus density of *Skn-1a*^-/-^ mice compared with that of *ChAT-eGFP* mice. Scale bars: 100 μm in A, 20 μm in B-D.

To clarify the impact of *Skn-1a* deficiency on the generation of Trpm5-microvillous cells, we quantified the microvillous cell density in the most superficial layer of the MOE of *Skn-1a*^-/-^ mice and compared it with the density obtained from the *ChAT*^*(BAC)*^*-eGFP* transgenic mice (*ChAT-eGFP*) as control. This quantification is possible because the MOE layer structure is largely unchanged in the *Skn-1a*^-/-^ mice. Because the epithelium in the lateral MOE is generally thinner, and because nuclei of *ChAT*/*Trpm5*-expressing microvillous cells often reside along with supporting cell nuclei [5], we focused on the dorsomedial region, where a majority of *ChAT*/*Trpm5*-expressing microvillous cells have their nuclei located in the most superficial layer, separated from the tightly packed nucleus layer of the supporting cells (Figure 6A; see also enlarged images from *ChAT-eGFP* and *Skn-1a*^-/-^ mice in B and C, respectively). We found that, on average, the densities of DAPI-stained nuclei and GFP-positive (*ChAT*/*Trpm5*-expressing) microvillous cells were 915 ± 67.6 nuclei/mm^2^ and 733 ± 38.2 cells/mm^2^ surface area, respectively, in the *ChAT-eGFP* mice (mean ± SD, n = 3). Thus, *ChAT*/*Trpm5*-expressing microvillous cells account for about 80% of the cells in the superficial layer in the dorsomedial region of the MOE (Figure 6D). Approximately 20% of the nuclei in the most superficial layer belong to cells with unknown identity. In *Skn-1a*^-/-^ mice, the number of DAPI-stained nuclei in the superficial layer of the dorsomedial region was drastically reduced, and the averaged nucleus density was 244 ± 49.5 nuclei/mm^2^ surface area (n = 3 mice; Figure 6E). Compared with the density obtained from the *ChAT-eGFP* mice (915 ± 67.6 nuclei/mm^2^), this is a 73% reduction in the density of superficial nuclei, comparable to the percentage of *ChAT*/*Trpm5*-expressing microvillous cells in the total cell (nuclei) count of the region. The reduction is statistically significant (Student’s *t*-test, p < 0.001). Since we did not observe apparent zonal variation in the density of *ChAT*/*Trpm5*-expressing microvillous cells in our previous studies [5,12], or in the present study, we believe these results are representative and that *Skn-1a* knockout diminishes the population of *ChAT*/*Trpm5*-expressing microvillous cells in the MOE. Thus, combined with the lack of Trpm5, villin, and ChAT expression in the *Skn-1a*^-/-^ mice, our results strongly indicate that Skn-1a functions as a critical regulator for the generation of Trpm5-microvillous cells in the MOE.

## Discussion

We found that *Skn-1a* is expressed in both *Mash1-*positive olfactory progenitors and Trpm5-microvillous cells in the MOE. Although *Skn-1a* is expressed in both cell lineages, the loss-of-function mutation of *Skn-1a* had differential impacts: grossly normal differentiation of OSNs and complete loss of Trpm5-microvillous cells.

In the absence of Skn-1a, OSNs differentiated normally except for a partial defective differentiation in a limited region of the MOE: ectoturbinate 2. Because only a small population of *Skn-1a*-expressing cells coexpressed *Mash1*, and most of *Mash1*-expressing progenitors did not coexpress *Skn-1a,* Skn-1a could not be a determining factor for OSN differentiation. Considering the partial penetrance of this minor phenotype (defective differentiation in ectoturbinate2), it could be due to a secondary effect of loss of Skn-1a function. However, we could not exclude the possibility that Skn-1a somehow interacts with Mash1 genetic pathways and might cause this phenotype in *Skn-1a*^-/-^ mice.

In contrast, none of the markers for Trpm5-microvillous cells were detectable in the *Skn-1a*^-/-^ MOE, and a drastic loss of microvillous cells was clearly demonstrated. Therefore, Skn-1a is not simply required for the expression of marker genes, but is necessary for generation of Trpm5-microvillous cells. Previous studies have shown that Skn-1a is essential for the generation and/or functional differentiation of chemosensory cells, such as sweet, umami, and bitter taste cells in taste buds and SCCs in nasal epithelium [13,14]. Both types of chemosensory cells share molecular characteristics of chemoreceptors, intracellular signaling molecules, and physiological functions to detect noxious substances [7,9,13,15]. Although they do not express taste-cell-like signaling molecules except for Trpm5 and ChAT [5,7,12], Trpm5-microvillous cells function as chemo- and thermo-sensitive cells by responding to certain chemical or thermal stimuli, and they release acetylcholine to modulate activities of neighboring supporting cells and OSNs [12]. It is intriguing that Skn-1a is commonly critical to generate these chemosensory cells.

There are at least three types of microvillous cell in the MOE [3]: two are Trpm5-microvillous cells, and one is a Trpm5-negative microvillous cell. Our quantitative analyses of the density of superficial cells showed that the reduction in the density of Trpm5-microvillous cells in *Skn-1a*^-/-^ mice is comparable to the percentage of *ChAT*/*Trpm5*-expressing microvillous cells, and that there are residual superficial cells, presumably non-Trpm5-microvillous cells. Currently, the identity of non-Trpm5-microvillous cells is unknown. The MOE, however, has a population of non-Trpm5-microvillous cells, called IP_3_ receptor type 3-expressing microvillous cells (IP_3_R3-microvillous cells) that express distinct cell markers, such as TRPC6, IP_3_R3, and PLC-β2 [2-5]. Double-label immunostaining against IP_3_R3 showed that immuno-signal of IP_3_R3 remained in *Skn-1a*^-/-^ mice, whereas that of Trpm5 was abolished, suggesting that one of remaining superficial microvillous cells in *Skn-1a*^-/-^ mice would be IP_3_R3-microvillous cells. (see Additional file 2: Figure S1). These indicate that Skn-1a is involved in the generation of Trpm5-microvillous cells but that its deficiency would not cause loss or expansion of non-Trpm5-microvillous cells; this differs from the case in taste buds, where Skn-1a regulates the fates of type II (sweet, umami, and bitter) and type III (sour) taste cells. In the microvillous cell lineages in the MOE, Skn-1a would not function to determine the lineage between Trpm5- and non-Trpm5-microvillous cells but would promote functional differentiation of Trpm5-microvillous cells. Further analysis of the function of Skn-1a in the olfactory epithelial cell lineages would provide us better understanding on the olfactory epithelial cell lineages.

## Conclusions

Here we show that in the MOE, *Skn-1a* is expressed mainly in *Trpm5-*expressing microvillous cells and is required for their generation in the MOE. Combined with previous observations, this study shows that Skn-1a is a critical transcription factor for generation and/or functional differentiation of several types of chemosensory cells, that is, sweet, umami, and bitter taste cells, SCCs, and Trpm5*-*microvillous cells in the nasal and oropharyngeal epithelium. It is possible that Skn-1a could be involved in generation of chemosensory cells in other epithelial tissues, such as brush cells in trachea and intestine. The expression and function of *Skn-1a* in those cell types will be investigated in future studies to reveal common molecular mechanisms of Skn-1a function in generation of closely related chemosensory cells.

## Methods

### Mutant mice

*Skn-1a/Pou2f3*-deficient mice (*Skn-1a*^-/-^) and *Mash1*-deficient mice (*Mash1*^-/-^) were generated as described elsewhere [13,16]. The *ChAT*^*(BAC)*^*-eGFP* transgenic (*ChAT-eGFP*) mice were kindly provided by Dr. M. I. Kotlikoff [17]. All mice used in this study were C57BL/6 background, and mutant and wild-type mice/embryos of either sex were used. For embryo staging, midday of the day of the vaginal plug was designated as embryonic day 0.5. The day of birth was designated postnatal day 0. All mouse studies were approved by the institutional animal experiment committees of University of Maryland, Baltimore County, of Monell Chemical Senses Center, and of Tokyo Institute of Technology and were performed in accordance with institutional and governmental guidelines.

### *In situ* hybridization

Probes for *Skn-1a*, *Trpm5*, *Plcb2*, *Gnat3*, *Tas1r3*, *Tas2r105*, *Tas2r108*, *Mash1*, *Ngn1*, *NeuroD*, *GAP43*, and *OMP* were prepared as previously described [13,14,18]. The MOE was cryosectioned coronally at 10 μm thick. Single- and two-color *in situ* hybridization was performed according to the method described previously [19,20]. For two-color *in situ* hybridization, the tyramide signal amplification-dinitrophenyl system (PerkinElmer) was used. The images were taken on an Olympus BX51 microscope with a DP71 digital CCD camera for bright-field images and a Leica SPE confocal microscope for fluorescent images.

### Quantitative analyses

To quantify the number of *Skn-1a-*, *Mash1*-, and *Trpm5*-expressing cells, every 10th coronal section (10 μm thickness) throughout the MOE was collected for *in situ* hybridization experiments, and the number of positive cells was counted. Experiments were conducted using three mice at P30, and the populations were calculated as the mean value.

### Immunohistochemistry

Immunohistochemistry was performed according to a previously described method using coronal cryosections of 10 μm thick [12,21]. The following primary antibodies and dilutions were used: goat anti-villin antibody (1:50; #sc-7672, Santa Cruz Biotechnology, Santa Cruz, CA), rabbit anti-Trpm5 antibody (1:500; #ACC-045, Alomone Labs, Jerusalem, Israel), goat anti-ChAT antibody (1:100; #AP144P, Millipore, Billerica, MA), mouse anti-IP_3_R3 antibody (1:500; #61312, BD Biosciences, San Jose, CA) with the Vector M.O.M. Immunodetection Kit (Vector Laboratories, Burlingame, CA). The following appropriate secondary antibodies were used: Alexa-546-conjugated anti-goat IgG antibody, Alexa-555-conjucated anti-goat IgG antibody (both from Invitrogen, Carlsbad, CA), and biotin-conjugated goat anti-rabbit IgG antibody (Vector Laboratories) with streptoavidin-Alexa-488 fluorescence (Invitrogen). We performed antigen-retrieval pretreatments in Target Retrieval Solution, pH 9.0 (Dako, Glostrup, Denmark) for 20 min at 80°C. The sections were coverslipped with Vectashield mounting medium with DAPI (Vector Laboratories) or Fluomount-G including DAPI for nuclear staining (Southern Biotechnology, Birmingham, AL).

### Quantitative analysis of the microvillous cell density

The density of the microvillous cells and nuclei in the most superficial layer of the MOE, which corresponds to the region above the nucleus layer of the supporting cells, was determined. Olfactory epithelial tissues from *ChAT-eGFP* control mice, in which *ChAT*/*Trpm5-*expressing microvillous cells are found throughout the entire MOE [12], and from *Skn-1a*^-/-^ mice were prepared as described previously [5,12], and a pair of noses from *ChAT-eGFP* and *Skn-1a*^-/-^ mice, respectively, were embedded side by side in a single block using Tissue-Tek OCT compound (Sakura Finetek, Torrance, CA). Every 20th coronal section (14 μm thickness) throughout the MOE was examined. Images of dorsal recess and septum region of the both sides of MOE in the sections were taken using an epifluorescence microscope (Olympus BX41, 10x objective) equipped with a CCD camera (QImaging Retiga 4000) and reconstituted using the MosaicJ plug-in of NIH Image J software. In the *ChAT-eGFP* mouse, both GFP-positive (GFP^+^; *ChAT*/*Trpm5*-expressing) microvillous cells and DAPI-stained nuclei in the most superficial layer of the MOE above the nuclei of supporting cells were counted manually. To avoid including supporting cells in the count, we counted only nuclei separated from the supporting cell nucleus layer, regardless the GFP expression. The surface area where the counting was conducted was determined by measuring the epithelial length using NIH Image J multiplied by the thickness of the section (14 μm). The density of both *ChAT*/*Trpm5*-expressing GFP^+^ cells and DAPI-stained nuclei of the MOE superficial layer was calculated using the number of cells or nuclei counted divided by the MOE surface area where the counting was conducted. Similarly, in *Skn-1a*^-/-^ mouse, DAPI-stained nucleus density of the MOE superficial layer was determined. Data were expressed in number of cells ± SD per mm^2^, and Student’s *t*-test was used to determine the statistical significance.

## Competing interests

The authors declare that they have no competing interests.

## Authors’ contributions

WL, IM, and JH designed and performed the research. TY, JY, MO, and AB performed *in situ* hybridization and immunohistochemistry. IA, TO, and W Luo performed quantitative analysis of the cell density. W Lin, IM, and JH wrote the manuscript. All authors read and approved the final manuscript.

## Supplementary Material

Additional file 1: Table S1 Summary of the quantification of *Skn-1a-, Trpm5-,* and *Mash1-*expressing cells in the MOE. The populations of *Skn-1a-, Trpm5-,* and *Mash1-*expressing cells in the MOE were quantified by *in situ* hybridization. This table summarizes the total numbers of cells counted in individual mice to calculate the populations in Figure 2D and E. Click here for file

Additional file 2: Figure S1 IP_3_R3 -positive microvillous cells remained in the *Skn-1a*^-/-^ MOE. Effects of *Skn-1a* deficiency on IP_3_R3 -positive microvillous cells were examined by immunostaining with an anti-Trpm5 (green) and an anti-IP_3_R3 (red) antibodies using coronal sections of the wild-type and *Skn-1a*^-/-^ MOE of adult mice. Trpm5-positive cells did not overlap with IP_3_R3-positive cells in the wild-type MOE. In the *Skn-1a*^-/-^ MOE, IP_3_R3-positive cells were observed, whereas no immunoreactive signal for Trpm5 was detected, suggesting that one of remaining superficial microvillous cells in the *Skn-1a*^-/-^ MOE would be IP_3_R3-microvillous cells, and Skn-1a is not essential to generate them. Scale bars, 10 μm. Click here for file
