# Supplementary material for: Skn-1a/Pou2f3 is required for the generation of Trpm5-expressing microvillous cells in the mouse main olfactory epithelium
Source: BMC Neurosci. 2014 Jan 16;15:13. doi: 10.1186/1471-2202-15-13 (PMC3901341; doi:10.1186/1471-2202-15-13)
Supplement: Additional file 1: Table S1 — Summary of the quantification of Skn-1a-, Trpm5-, and Mash1-expressing cells in the MOE. The populations of Skn-1a-, Trpm5-, and Mash1-expressing cells in the MOE were quantified by in situ hybridization. This table summarizes the total numbers of cells counted in individual mice to calculate the populations in Figure 2D and E. [file 1471-2202-15-13-S1.pdf]

Supplemental Table S1

Summary of the quantification analyses of *Skn-1a-, Trpm5-, and Mash1*-expressing cells in the MOE.

	Mouse ID	S384	S656	S660	Average	SD
Number of cells	Skn1a+ cells	787	199	214	400	335
	Trpm5+ cells	1395	731	378	835	516
	Skn1a+Trpm5+ cells	603	144	180	309	255
Population (%)	Skn1a+Trpm5+ / Skn1a+	76.6%	72.4%	84.1%	77.7%	5.9%
	Skn1a+ Trpm5+ / Trpm5+	43.2%	19.7%	47.6%	36.8%	15.0%

	Mouse ID	S384	S656	S660	Average	SD
Number of cells	Skn1a+ cells	566	290	272	376	164.8
	Mash1+ cells	2963	2493	1269	2242	874.5
	Skn1a+ Mash1+ cells	29	30	26	28	2.1
Population (%)	Skn1a+ Mash1+ / Skn1a+	5.1%	10.3%	9.6%	8.3%	2.8%
	Skn1a+ Mash1+ / Mash1+	1.0%	1.2%	2.0%	1.4%	0.56%
