# Supplementary material for: Skn-1a/Pou2f3 is required for the generation of Trpm5-expressing microvillous cells in the mouse main olfactory epithelium
Source: BMC Neurosci. 2014 Jan 16;15:13. doi: 10.1186/1471-2202-15-13 (PMC3901341; doi:10.1186/1471-2202-15-13)
Supplement: Additional file 2: Figure S1 — IP3R3 -positive microvillous cells remained in the Skn-1a-/- MOE. Effects of Skn-1a deficiency on IP3R3 -positive microvillous cells were examined by immunostaining with an anti-Trpm5 (green) and an anti-IP3R3 (red) antibodies using coronal sections of the wild-type and Skn-1a-/- MOE of adult mice. Trpm5-positive cells did not overlap with IP3R3-positive cells in the wild-type MOE. In the Skn-1a-/- MOE, IP3R3-positive cells were observed, whereas no immunoreactive signal for Trpm5 was detected, suggesting that one of remaining superficial microvillous cells in the Skn-1a-/- MOE would be IP3R3-microvillous cells, and Skn-1a is not essential to generate them. Scale bars, 10 μm. [file 1471-2202-15-13-S2.pdf]

Additional file 2

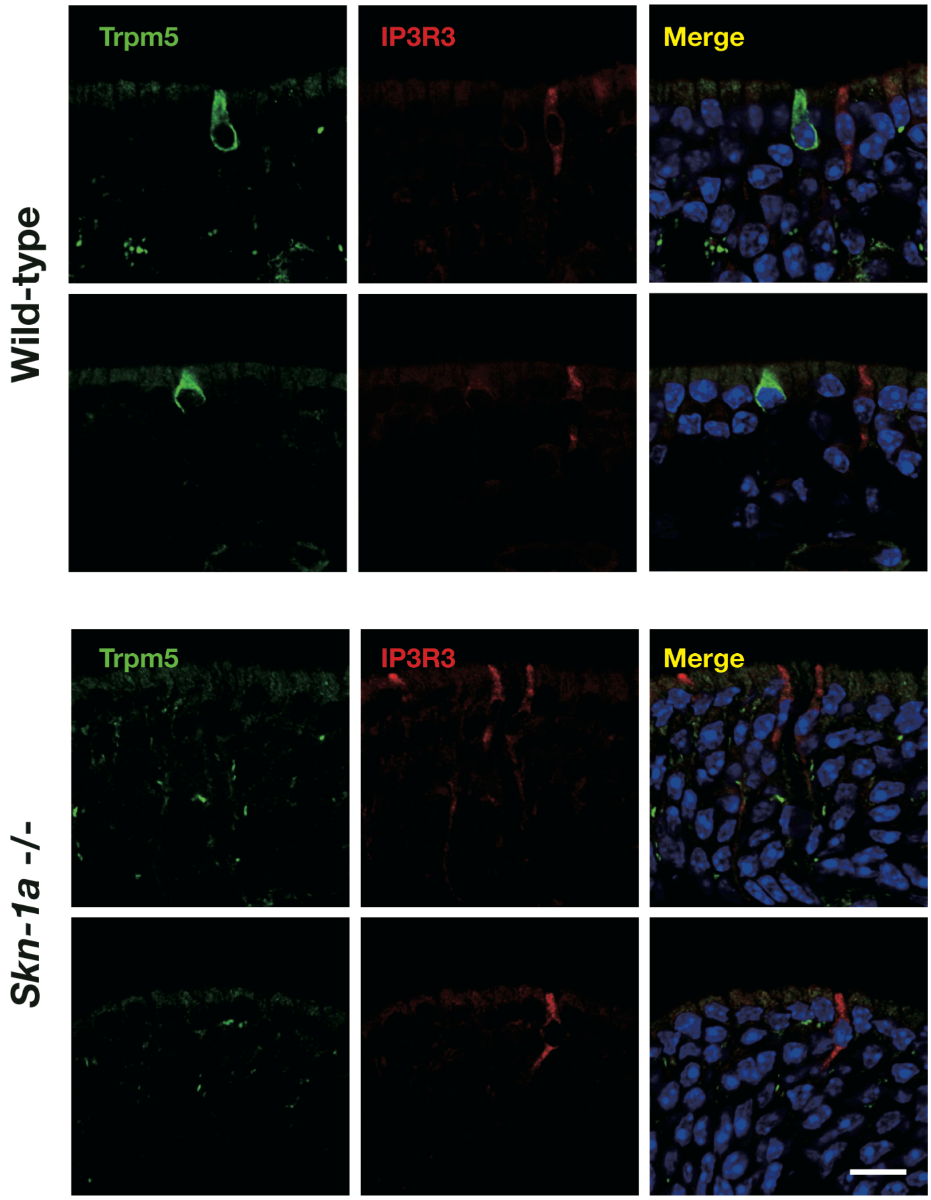

Supplemental Figure S1. IP_3R3 -positive microvillous cells remained in the *Skn-1a*$^{-/-}$ MOE.

Coronal sections of the wild-type and *Skn-1a*$^{-/-}$ MOE of adult mice were immunostained with an anti-Trpm5 antibody (green) and an anti-IP_3R3 (red). Trpm5-positive cells did not overlap with IP_3R3-positive cells in the wild-type MOE. In the *Skn-1a*$^{-/-}$ MOE, IP_3R3-positive cells were observed, whereas no immunoreactive signal for Trpm5 was detected, indicating that IP_3R3-positive microvillous cells are distinct from Trpm5-microvillous cells and Skn-1a is not a critical factor to generate IP3R3-microvillous cells in the MOE. Scale bars, 10 µm.
